# Socio-Demographic Predictors of Willingness to Pay for Premium of National Health Insurance: A Cross-sectional Survey of Six Districts in Sierra Leone

**DOI:** 10.34172/ijhpm.2021.50

**Published:** 2021-05-26

**Authors:** Peter Agyei-Baffour, Abraham Isiaka Jimmy, Peter Twum, Deborah Larbie, Kwabena Anarfi Boateng, Ibrahim Kwaku Duah, Abdul Bangura, Hassan Milton Conteh

**Affiliations:** ^1^Department of Health Policy Management and Economics, School of Public Health, College of Health Sciences, Kwame Nkrumah University of Science and Technology (KNUST), Kumasi, Ghana.; ^2^University of Makeni (UNIMAK), Makeni, Sierra Leone.; ^3^College of Nursing and Midwifery, Nalerigu, Ghana.; ^4^Sierra Leone Ministry of Health and Sanitation, Makeni, Sierra Leone.; ^5^Medicine San Frontières (MSF), Makeni, Sierra Leone.

**Keywords:** Predictors, Willingness, Premium, Health Insurance, Districts, Sierra Leone

## Abstract

**Background:** The government of Sierra Leone introduced Social Health Insurance Scheme as a measure to remove financial barriers that beset the people in accessing health to ensure universal coverage. Under this policy, the citizens were encouraged to subscribe to the scheme to avoid out of pocket payment for healthcare at the point of use. This study was conducted to find out the predictors of willingness among the people to pay for health insurance premium.

**Methods:** A cross-sectional study design was employed in six selected districts in Sierra Leone. Quantitative data was collected for this study through the use of semi-structured questionnaire with a sample size of 1185 respondents. Data was analysed into descriptive and inferential statistics using the contingent valuation model. Statistical analysis was run at 5% significant level using Stata version 14.0 software.

**Results:** The results showed that majority of the respondent are willing to join and pay a monthly premium of Le 10 000 (US$1.03) with an estimated mean contribution of about Le 14 089 (US$1.44) and the top five predictors of willingness to pay (WTP) were household monthly income, age, district of resident, gender, and educational qualification.

**Conclusion:** The findings on predictors of WTP premium of Sierra Leone National Social Health Insurance (SLeNSHI), suggests that the socio-demographic characteristics of the population are important in premium design and payment. Efforts at improving the socio-economic statuses of the population could be helpful in premium design and payment.

## Background

**Key Messages**

**Implications for policy makers**

- It will guide the relevant government ministries to make inform decision on healthcare financing.
- It will provide policy direction to policy-makers.
- It will lead to bottom-up approach to policy decision-making.
- It will guide policy-makers to address the concerns of the people they serve.
- It will bring out the weakness as well as strength in the health system.

**Implications for the public**

It is generally accepted that the development of every country, to a very large extent, depends on the health of its citizens. However, access to healthcare in developing countries such as Sierra Leone, is beset with financial restrains such as out of pocket payment which push families into poverty. Therefore, the implementation of health insurance scheme will remove or at worse minimise financial burden on the people at the time of seeking healthcare. As a result, the people will have access to healthcare to improve their health and become more productive to enhance economic development of the country.

The role governments in developing countries play in funding improvements in the health of their citizens has taken centre stage of discussion as the international community deliberate on the Sustainable Development Goals and health priorities more generally in the post-Millennium Development Goals era.^1,2^ The growth in gross domestic product of most of these developing countries in recent time couple with their abilities to roll out more comprehensive health insur­ance schemes with sufficient tax revenue to fund them, keeps on pointing out that paying attention to these sources may contribute to a more sustainable and accountable funding environment for health in developing countries.^3^ This will aid individuals to use the needed services without causing them any financial impoverishment. Healthcare financing systems that aid the attainment of universal health coverage can improve the provision and effective use of an efficient mix of both personal and non-personal health services.^4,5^

 One way to finance healthcare is through social health insurance.^5^ The idea of Social health insurance schemes is mostly understood as health insurance schemes delivered by governments to its citizens, particularly, low and middle-income populaces. Social health insurance is capable of pooling the financial risk of its members on one side, and the premium contributions of households, government, and enterprises on the other. Whenever general healthcare coverage is to be funded through insurance, the financial risk pool needs to portray certain characteristics; (*i*) involuntary contributions to the risk pool so as to thwart the rich and healthy from opting out; (*ii*) the risk pool should have great numbers of people, as pools with a fewer number cannot broaden the risk satisfactorily and are too small to handle large health costs; and (*iii*) where the majority of the members are poor, pooled funds will generally be subsidised from government revenue.^6^

 One unique feature of social health insurance is that it can improve the welfare through improvement of the health status and the maintenance of non-health consumable goods by ensuring that health expenses are smoothened over time and hence there is no substantial drop in household supply.^3,7,8^ However, this will depend much on people’s willingness to pay (WTP). In a study conducted in Sierra Leone by Jofre-Bonet and Kamara on WTP for health insurance in the informal sector, they found out the average WTP for the health insurance is Le 20 237.16 (US$3.6) per adult but it ranges from about Le 14 000 (US$2.5) to about Le 35 000 (US$6.2) depending on region, occupation, household and respondent characteristics.^9^

 The Ministry of Health and Sanitation of Sierra Leone is the country’s main healthcare provider and aims to improve and upgrade the health status of all citizens.^10^ Based on the agenda for change by the government and the 2011–2015 health sector strategic plans, the Free Healthcare Initiative was implemented in April 2010 to provide free access to healthcare services for lactating mothers, pregnant women, and children under the age five. The initiative focuses mainly on essential healthcare packages that would be delivered with no cost attached for the targeted individuals during the point of service delivery to significantly improve the maternal and child health indices.

 Healthcare coverage under the Free Healthcare Initiative does not necessarily cover the rest of the population who often face high and catastrophic user fees. The majority of Sierra Leone’s populations are required to pay user fees to access healthcare. To reverse this, there is an interest from the government of Sierra Leone to explore alternatives that would ensure access to quality, equitable, and affordable healthcare for the Sierra Leone population through social health insurance scheme to attain the goal of UHC. However, less is known on the predictors of WTP for premium of the National Health Insurance in Sierra Leone.

 This study was therefore undertaken to identify the socio-demographic predictors of WTP for premium of the Sierra Leone National Social Health Insurance (SLeNSHI) to serve as an evidence base for policy-makers in formulating policies relating to adequate healthcare payment that can improve efficiency and effectiveness for countries struggling with their healthcare financing system.

## Methods

 A cross-sectional analytical study was used to find out the predictors of WTP for health insurance premium among 1185 household heads from a total household population of 3 587 724 in six districts; Kono (505 491), Bo (574 026), Koinadugu (408 687), Bombali (605 741), Western area Urban (1 050 711), and Western Area Rural (443 068) in Sierra Leone.

 In the first-place, systematic sampling method was used to select the houses based on the data from the Sierra Leone Statistics Office. We used the constant term (Kth term), to divide the study population by the sample size (3 587 724/1185 = 3028**), **out of every 3028 house, the 3028^th^ was selected. If for any reason the household heads were not around, the data collector automatically enrolled the immediate household. In a house where there is only one household, a convenient sampling was used to select the household head. Where there were more than one household, a simple random (lottery) was used to select one to take part in the study. The simple random method was used to ensure that households heads have equal chance of being selected for the study.

###  Sample Size Determination 

 The sample size was estimated from the study population using a sample size calculator version 2.0.2 by Relief Applications. Using a 95% confidence level and a precision rate of 0.05, this gives a sample size of 1185 household heads from the total household population of (3 587 724).

###  Data Analysis

 Data was obtained from household head through the use of semi-structured questionnaire after it was pre-tested. The data was analysed using statistical package STATA version 14.0. Logistics regression analysis was run to determine possible association between socio-demographic characteristics of respondents and WTP for premium for the proposed SLeNSHI. Based on a logistic regression analysis, WTP for premium may be determined or predicted by the district where individuals reside, location of residence, age, gender, marital status, religion, monthly income, and educational qualification. All statistical tests were conducted at the level of significance *P*< .05.

###  Willingness to Pay Valuation Model

 The WTP for the proposed SLeNSHI was estimated according to the contingent valuation model. The contingent valuation model is considered to be an important survey model to bring out the WTP for services and public goods in developing countries in a hypothetical market that can generate estimates for unimplemented public policies or projects^11,12^ as in the case of this study. In contingent valuation, the proposed market is clearly explained to participants (contingency) and a number of questions are asked to point out the maximum amount the respondent would be willing to contribute. As a result of its flexibility, many studies have used the contingent valuation method worldwide to estimate the WTP,^13^ which includes Northern Ethiopia, China,^12^ Ghana^11^ and South Sudan.^14^ This method has been widely used in different areas of studies as in environmental, cultural, health, tourism and transport.^15^ The contingent valuation has been stated as a leading preference method rather than a revealed preference method for identifying directly the overall economics values; both use value and non-use values of a product or service in a form of opinion.^15^

 In general, WTP can be more or less elicited in three main approaches using contingent valuation. The first approach is through open-ended questions. Under this approach, the respondent is questioned on how much he/she will be willing to pay for a previously described good or service. The next approach is by using payment cards; in this approach, the participants are presented with a couple of possible payment amounts options and one is chosen nearest to their valuation. The final model is to use dichotomous choice questions. In this approach, the individual are asked (are you willing to pay X, Yes or No?) after been presented with a described hypothetical amount^16^ the last method is the one used in this study.

 Under this dichotomous method, the respondent is given two different amounts; hence the second amount is dependent on the feedback from the first charges. If the participant responds ‘yes’ to the first charge, a second charge twice higher than the first would be presented. If the participant responded ‘no’ to the first charge, a second lower charge than the first would be presented.^11,16,17^ In an assumption, it is possible to estimate the WTP using the linear econometric model as follows:


(1) $$WTP_{i}\left(z_{i},u_{i}\right)=z_{i}\beta +u_{i}$$

The *z*_i_ represents a vector of independents variable, *β* is a vector of parameters and *u*_i_ is the term of error assumed to be disseminated randomly and independently with zero as mean and constant variance, *σ*^2^.

 Let the first charge number be “*q*_1_” and the second charge“*q*_2_” and then the person will have the following probability result:

1. When the individual answers ‘yes’ to the first question and ‘no’ to the second question, then q_2_ > q_1_. In such an instance, we then deduce that q_1_ ≤ WTP < q_2_.
2. When the individual answers ‘yes’ to the first question and ‘yes’ to the second one, then q_2_ ≤ WTP < ∞.
3. When the individual answers ‘no’ to the first question and ‘yes’ to the second one, then q_2_ < q_1_. In such a case, we have q_2_ ≤ WTP < q_1_.
4. When the individual answers ‘no’ to the first and second questions, then we have 0 < WTP < q_2_.

Therefore, the probability of each of the above four cases are stated as:


(2) $$\begin{array}{l} 1.\;\mathrm{Pr}\left(q_{1}\leq WTP<q_{2}\right)\\ \;=\mathrm{Pr}\left(\frac{q_{1}-{z^{\prime }}_{i}\beta }{\sigma }\leq \frac{u_{i}}{\sigma }<\frac{q_{2}-{z^{\prime }}_{i}\beta }{\sigma }\right)\\ \;=\Phi \left({z^{\prime }}_{i}\frac{\beta }{\sigma }-\frac{q_{1}}{\sigma }\right)-\Phi \left({z^{\prime }}_{i}\frac{\beta }{\sigma }-\frac{q_{2}}{\sigma }\right) \end{array}$$

(3) $$\begin{array}{l} 2.\;\mathrm{Pr}\left(WTP>q_{1},WTP>q_{2}\right)\\ \;=\Phi \left({z^{\prime }}_{i}\frac{\beta }{\sigma }-\frac{q_{2}}{\sigma }\right) \end{array}$$

(4) $$\begin{array}{l} 3.\;\mathrm{Pr}\left(q_{2}\leq WTP<q_{1}\right)\\ \;=\Phi \left({z^{\prime }}_{i}\frac{\beta }{\sigma }-\frac{q_{2}}{\sigma }\right)-\Phi \left({z^{\prime }}_{i}\frac{\beta }{\sigma }-\frac{q_{1}}{\sigma }\right) \end{array}$$

(5) $$\begin{array}{l} 4.\;\mathrm{Pr}\left(WTP<q_{1},WTP<q_{2}\right)\\ \;=1-\Phi \left({z^{\prime }}_{i}\frac{\beta }{\sigma }-\frac{q_{2}}{\sigma }\right) \end{array}$$

In the approximation of *β* and *σ* was grounded on the supreme likelihood technique. The linear function requires to be exploited to identify the factors of the model is stated below:


(6) $$\sum_{i=1}^{N}\left[\begin{array}{l} d_{i}^{yn}\ln\left(\Phi \left({z^{\prime }}_{i}\frac{\beta }{\sigma }-\frac{q_{1}}{\sigma }\right)-\Phi \left({z^{\prime }}_{i}\frac{\beta }{\sigma }-\frac{q_{2}}{\sigma }\right)\right)\\ +d_{i}^{yy}\ln\left(\Phi \left({z^{\prime }}_{i}\frac{\beta }{\sigma }-\frac{q_{2}}{\sigma }\right)\right)+d_{i}^{ny}\ln\left(\begin{array}{l} \Phi \left({z^{\prime }}_{i}\frac{\beta }{\sigma }-\frac{q_{2}}{\sigma }\right)\\ -\Phi \left({z^{\prime }}_{i}\frac{\beta }{\sigma }-\frac{q_{1}}{\sigma }\right) \end{array}\right)\\ +d_{i}^{nn}\ln\left(1-\Phi \left({z^{\prime }}_{i}\frac{\beta }{\sigma }-\frac{q_{2}}{\sigma }\right)\right) \end{array}\right]$$

Where $\;d_{i}^{yn},\;d_{i}^{yy},\;d_{i}^{ny},\;d_{i}^{nn}\;$ represent binary variables with values of one representing the occurrence of that outcome for each individual and zero for its not occurring. Each and every respondent tends to add the likelihood function to the logarithm in just one of its four measures. Therefore one can openly obtain β and *σ* when the WTP is estimated. Hence the household WTP for the national health insurance can be specified as:


WTP=Φ+σq+βz+u

Where ‘q’ means the last charge level that the responded was presented with, ‘z’ represents the socioeconomic factor and ‘u’ represents the random variable accounting for unobserved factors, and Φ, σ and β are the estimated parameters. The empirical formula for the above equation is then formulated as:


$$WTP=\Phi +\sigma q+\beta_{1}\;AGE+\beta_{2}\;GEN+\beta_{3}\;EDU+\beta_{4}\;MARIT+\beta_{5}\;OCC+\beta_{6}\;RESI+\beta_{7}\;MINC$$

AGE represents age of respondents, the GEN represents gender of participants, EDU means educational level of respondents, MARIT means marital status of respondent, OCC represents occupation of participant, RESI is the residential location of respondent and MINC is the average monthly income of participant.

## Results

###  Socio-Demographic Characteristics of Household Heads

 The socio-demographic characteristics of respondents; district of household head, location, age, gender, marital status, religion, occupation, monthly income, and educational qualification are presented in Table 1. From the findings, majority (29.28%) of household heads were from Western Area Urban district. In terms of location of household heads in the district, more than half (69.28%) were from the urban areas. The data reveals that most (43.63%). Household heads are within the age bracket of 30-39 years. Majority of the household heads, 67.76% were males. A vast majority of the respondents (72.24%) are married. Christianity was practice by a little over half (52.66%) of the respondents. Regarding occupation, most 62.95% of the respondents are informal workers. On household heads’ monthly income, (mean = Le 1 069 945, SD = Le 1 307 152) the majority of them (37.47%) are earning less than Le 500 000. Again majority 39.92% of the household heads have tertiary education.

**Table 1:** Socio-Demographic Characteristic of Household Heads

**Variables**	**No. (n = 1185)**		**%**	
**District of household heads**				
Kono	167		14.09	
Bo	190		16.03	
Bombali	200		16.88	
Koinadugu	135		11.39	
Western Area Urban	347		29.28	
Western Area Rural	146		12.32	
**Location of household heads**				
Rural area	364		30.72	
Urban area	821		69.28	
**Age categories of household heads (y)**				
20-29	105		8.86	
30-39	517		43.63	
40-49	354		29.87	
50-59	177		14.94	
60+	32		2.70	
Female	382		32.24	
Male	803		67.76	
**Marital status of household heads**				
Single	161		13.59	
Married	856		72.24	
Divorced	101		8.52	
Widow	67		5.65	
**The religion of household heads**				
Islam	561		47.34	
Christianity	624		52.66	
**Occupation of household heads**				
Informal	746		62.95	
Formal	439		37.05	
**Categories of household head monthly income**	**No.**	**%**	**Mean**	**SD**
<Le 500 000	444	37.47	1069.94	1307.15
Le 500 000-1 000 000	418	35.27		
> Le 1 000 000	323	27.26		
**Educational qualification of household heads**				
No formal education	306		25.82	
Primary school education	158		13.33	
Secondary school education	248		20.93	
Tertiary education	473		39.92	
**Awareness of SLeNSHI implementation**				
No	904		76.29	
Yes	281		23.71	
**WTP and join the SLeNSHI by household heads**				
No	67		5.65	
Yes	1118		94.35	
**Amount willing to contribute in categories in Leones**	**No. ** **(n = 1118)**	**(%)**	**Mean**	**SD**
1000-9000	338	30.23	14 089.45	18 690.41
10 000-19 000	503	44.99		
20 000-29 000	161	14.40		
30 000-39 000	49	4.38		
40 000+	67	5.99		
**Willingness for percentage contribution from monthly Income**				
No	537		48.03	
Yes	581		51.97	
**Total**	**1118**		**100**	
**Percent of monthly income willing to contribute by formal workers (n = 581)**	**No.**	%	**Mean**	**SD**
1%	136	23.41	3.03	1.80
2%	165	28.40		
3%	61	10.50		
4%	29	4.99		
5%	178	30.64		
8%	7	1.20		
10%	5	0.86		

Abbreviations: WTP, willingness to pay; SLeNSHI, Sierra Leone National Social Health Insurance.

 Regarding the willingness to join the proposed scheme, almost all (94%) of the respondents were willing to join and pay the premium. The amount household heads were willing to contribute was analysed in ranges, it shows that (mean = Le 14 089.45; SD = Le 18 690.41), those that were willing to contribute Le 10 000-19 000 accounted for the highest (44.99%).

###  Chi-square Test of Independent/Relationship

 The chi-square test was conducted to ascertain the relationship between the level of WTP premium of the proposed scheme and socio-demographic characteristics. From the analysis, it statistically shows that, there is a significant relationship between the WTP premium for the proposed SLeNSHI and the following variables; district in which the individual stays, marital status, religion, occupation, monthly income, educational qualification and awareness on SLeNSHI implementation as shown in Table 2.

**Table 2:** Chi-square Test of Independent/Relationship

**Variables**	**No**	**Yes**	**No + Yes**	**No**	**Yes**	***P*** ** Value**
	** N**			** %**		
District of respondents	67	1118	1185	5.7	94.3	.001
Kano	0	167	167	0.0	100.0	
Bo	2	188	190	1.1	98.9	
Bombali	12	188	200	6.0	94.0	
Koinadugu	44	91	135	32.6	67.4	
Western area rural	3	143	146	2.0	98.0	
Western area urban	6	341	347	1.7	98.3	
Location of household	**67**	**1118**	**1185**	**5.7**	**94.3**	.021
Rural area	16	348	364	4.3	95.7	
Urban area	51	770	821	6.2	93.8	
Age of respondents (y)	**67**	**1118**	**1185**	**5.7**	**94.3**	.042
20-29	5	100	105	4.7	95.3	
30-39	32	485	517	6.2	93.8	
40-49	18	336	354	5.1	94.9	
50-59	8	169	177	4.6	95.4	
60+	4	28	32	12.6	87.4	
Gender of respondents	**67**	**1118**	**1185**	**5.7**	**94.3**	.013
Female	16	366	382	4.2	95.8	
Male	51	752	803	6.3	93.7	
Marital status	**67**	**1118**	**1185**	**5.7**	**94.3**	.002
Single	11	150	161	6.8	93.2	
Married	44	812	856	5.1	94.9	
Divorced	1	100	101	0.9	99.1	
Widow	11	56	67	16.4	83.6	
Religion	**32**	**1153**	**1185**	**2.8**	**97.2**	.001
Islam	24	537	561	4.3	95.7	
Christianity	8	616	624	1.3	98.7	
Occupation of household heads	**38**	**1147**	**1185**	**3.3**	**96.7**	.004
Informal worker	32	714	746	4.3	95.7	
Formal worker	6	433	439	1.4	98.6	
Monthly income of household heads (Le)	59	988	1047	5.6	94.4	.001
<500 000	26	366	392	6.8	93.2	
500 000-1 000 000	29	340	369	7.9	93.1	
>1 000 000	4	282	286	1.4	98.6	
Educational qualification	24	1161	1185	2.1	97.9	.002
Non-formal education	5	301	306	1.6	98.4	
Primary school education	6	152	158	3.8	96.2	
Secondary school education	5	243	248	2.0	98.0	
Tertiary education	8	465	473	1.7	98.3	
Awareness of SLeNHIS implementation	**67**	1118	1185	5.7	94.3	.014
No	64	840	904	7.1	7.1	
Yes	3	278	281	1.1	98.9	

Abbreviation: SLeNSHI, Sierra Leone National Social Health Insurance.

###  Predictors of Willingness to Pay Premium of SLeNSHI

 In this sub-section, the analysis in Table 3 were based on the contingent valuation model which is used for estimating WTP for services and public goods in developing countries like Sierra Leone. This analysis was done to identify possible socio-economic and demographic predictors of WTP for premium for the proposed SLeNSHI. Based on a logistic regression analysis, WTP for premium may be determined or predicted by the district where individuals reside, location of residence, age, gender, marital status, religion, monthly income, and educational qualification. For instance, people staying in the Koinadugu district were 95.8% less likely (odds ratio [OR] = 0.04, 95% CI = 0.01-0.19) willing to pay for the proposed SLeNSHI as compared to those in Kono district. Those residing in urban areas were 60.4% less likely (OR = 0.40, 95% CI = 0.16-0.99) WTP as compared to those in rural areas. Individuals of 60 years and above were 98.3% less likely (OR = 0.02, 95% CI = 0.00-0.16) WTP as to those within 20-29 years. As compared to females, males were 68% less likely (OR = 0.32, 95% CI = 0.13-0.84) WTP. The WTP of widows were 85% less as compared to those are single (OR = 0.15, 95% CI = 0.04-0.65). More so Christians were three times more likely (OR = 3.06, 95% CI = 1.42) willing to pay as compared to Muslims. Comparing to those earning less than Le 500 000 as monthly income, those earning more than Le 1 000 000 as monthly income have a higher odds of WTP (OR = 33.61, 95% CI = 6.74-167.66). Lastly, having any form of education was statistically associated with higher odds of WTP for the proposed SLeNSHI as compared to those with non-formal education.

**Table 3:** Logistic Regression of Socio-Demographic Predictors of WTP Premium

**Variables**	**OR**	***P*** ** Value**	**95% CI**
District of respondent			
Kono	Ref		
Bo	7.717772	.007	(0.85-70.19)
Bombali	0.6767063	.063	(0.14-3.31)
Koinadugu	0.042064	**.001**	(0.01-0.19)
Western Area Urban	2.909876	.221	(0.53-16.09)
Location of respondent			
Rural area	Ref		
Urban area	0.3963123	**.048**	(0.16-0.99)
Age of respondents (y)			
20-29	Ref		
30-39	0.5471078	.357	(0.15-1.97)
40-49	1.000478	.999	(0.25-4.02)
50-59	0.247084	.085	(0.05-1.22)
60+	0.0177635	**.001**	(0.01-0.16)
Gender			
Female	Ref		
Male	0.3244843	**.002**	(0.13-0.84)
Marital status			
Single	Ref		
Married	0.5753017	.271	(0.22-1.54)
Divorced	9.336303	.066	(0.87-100.69)
Widow	0.1531334	**.011**	(0.04-0.65)
Occupational status			
Informal workers	Ref		
Formal worker	1.65385	.039	(0.53-5.21)
Religion of respondent			
Islam	Ref		
Christianity	3.060239	**.004**	(1.42-6.59)
Monthly income (Le)			
<500 000	Ref		
500 000-1 000 000	1.960328	.128	(0.82-4.66)
>1 000 000	33.61017	**.001**	(6.74-167.66)
Educational qualification			
Non-formal education	Ref		
Primary school education	0.9086821	.879	(0.26-3.13)
Secondary school education	2.89669	**.027**	(1.13-7.42)
Tertiary education	0.5862888	.378	(0.18-1.92)
Constant	89.71507	.001	(11.16-720.93)

Abbreviations: WTP, willingness to pay; OR, odds ratio.

## Discussion

 Financial barrier remains important access barriers in most developing and middle-income countries. Consequently, countries including Sierra Leone are directing their much of the efforts towards finding sustainable financial solutions to address the acute access to healthcare challenge. Social health insurance has been sound appropriate and feasible to improve access to health. We set out to investigate the WTP premium in the proposed SLeNSHI to inform implementation.

 Socio-demographic characteristics such as place of residence, age, marital status, education, employment, sex, income and religion are known to influence WTP for health insurance premium. This study attests to this assertion as urban area, age, sex (male), married, income levels and educational levels were found to have association with the WTP health insurance premium. This could be attributed to reasons such as, but not limited to, the household heads hope to benefit from the scheme as the government will take care of their high health related expenditure. They may also belief that it will help them and their family members to access healthcare at a reduced cost or safe them from direct out-of-pocket payment. In addition, it will help them to have better or quality healthcare service. They also believed that it would be helpful to them in times of emergency, especially when money may not be readily available for seeking healthcare due to the uncertainty of disease occurrence. This finding is in line with a study conducted by Mekonne et al^17^ andGlobal Burden of Disease Health Financing Collaborator Network,^18^ among civil servants in the City of Mekelle, the Northern part of Ethiopia stated; WTP has been correlated significantly with age, educational level, and household income.

 This study further found that majority of the respondents were not aware of the implementation of the proposed scheme. This may be due to inadequate public sensitization regarding the propose scheme. This is in contrast with the findings of Basaza et al,^14^ who found more than half of the public servants in Juba City being aware of NHIF. However, the findings is supported by a study in Nigeria,^19^ which found less than half of the participants being aware of community-based health insurance.

 A majority of participants were extremely willing to join the scheme and to pay the premium, only a few disagree to join the scheme. Reasons for willing to join was that they would benefit from the scheme as the government would take care of their high health-related expenditure, help their family members to access healthcare by reducing their direct pocket payment, they also believed that it would be helpful to them in terms of emergency when money is not readily available in seeking healthcare due to the uncertainty of disease occurrence and would be more beneficial to the poor. Those who said they were not willing to join the scheme perceived that premium contribution might be high as they have other family responsibilities to address. Some respondents also stated lack of trust and confidence in the government for the sustainability of the program and believe that corruption could collapse the scheme.

 A significant percentage of respondents said they were willing to contribute a mean of Le 14 089.45; 95% CI (12 992.67-15 186.22), as premium. According to literature,^20-22^ one key important principle in social health insurance scheme is that premium for formal workers is mostly charged as a percentage of monthly income, which is to ensure that contributions are made according to ability in ensuring that access to health service is based on need. Based on this principle it is obvious the outcome of this study reveals that more than half of the formal workers are willing to contribute to the proposed scheme. The mean average percentage contribution from monthly income is considered to be slightly above 3%. These findings are slightly different from what was found in a study by Basaza et al^14^ which saw that more than two-thirds (67.8%) of those willing to pay could pay up to 5% of their total monthly income, 22.9% could pay up to 10% and the rest could pay 25%.

 This is possibly because the monthly income-earning of people is low and the level of poverty is an important factor as well. Moreover, the premium respondents are willing to contribute is inconsistent with the proposed 4% premium contribution from formal workers as per the proposed National health insurance scheme in Uganda^23^ as cited by Carrin et al.^21^ This may imply that the resident in Sierra Leone are not willing to pay any amount that is way beyond the said mean contributions especially the informal workers. Furthermore, it implies that the funds that will be available for the scheme when established will be significantly low, except there are other sources of funds which could be from the central government, corporate bodies and external sources.

 From the contingent valuation model,^24^ the result shows that major socio-demographic and economic factors such as the district where the respondent lives, location of residence, age, gender, marital status, educational qualification, and monthly income are associated with the healthcare. A household with higher income is likely to have a high WTP as compared with those with low monthly income. This finding is not surprising as it corroborate a study on WTP for Community-based health insurance in Nigeria.^5,12^ On the contrary, this outcome was in contrast with the findings from Basaza et al^14^ study on willingness to contribute to national health insurance funds among public servants in Juba City, which reported that monthly income was not a significant predictor of WTP.

 Individuals with any other form of educational qualification as compared to non-formal education are more willing to pay. This finding is similar to the findings of Boateng et al,^11^ Dror et al,^25^ and Sydavong et al^26^ which looked at predictors of healthcare for improved solid waste management in Ghana and found out that education was significantly associated with WTP. This study shows that older age (60+ years) is negatively correlated to WTP, older age respondents are willing to pay less than those in the younger age brackets. This was in line with other studies^27^ but contrary to a study by Emodi et al^28^ which stated that aged individuals with higher health risk are willing to pay more. This study found out that awareness of resident is a predictor of WTP for the proposed SLeNSHI which is consistent with the findings of other studies^14^ which also found that WTP increases with awareness.

###  Strengths and Weakness

 The study was one of the few studies done on the proposed SLeNSHI, characterized the population, covered more rural areas and focused on the WTP for the proposed social health insurance scheme. However like any cross sectional study design, it is possible the results may have been affected by social desirability, sample variability and recall bias which could have under or overestimated the income and amount of money respondents said they are willing to pay as premium. These notwithstanding, the study upheld the measures to ensure validity and reliability by employing random sampling, pretesting of tools, and making statistical adjustments such as design effects and non-response rates and subjecting the study protocols to Institutional Review of Board-Office of Sierra Leone Ethics and Scientific Review Committee of Sierra Leone which are consistent with best scientific practice. We strongly believe that these measures reduced the errors to the barest level and would not have any dire effects on the policy utility of the findings.

## Conclusion

 Majority of the household heads were married, Christians, had tertiary education, informal workers with monthly income of less than Le 500 000. Households heads were willing to pay for the health insurance premium due to its expected benefits. Majority of them were willing to contribute a mean of 14 089 Leones (45 cent), and 3.03% of their income as their yearly premium. Based on the contingent valuation model, the top five predictors of WTP were household monthly income, age, district of resident, gender, and educational qualification.

## Acknowledgements

 We thank the data collection team from University of Makeni for their job well done. Our sincere gratitude also goes to the Sierra Leone Ministry of Health and Sanitation for giving us access to their data for the purpose of this study.

## Ethical issues

 Ethical approval was sought and received from the ethical review board from Sierra Leone Ethics and Scientific Review Committee. An inform consent form was issued out to respondents to seek their knowledge and to assure them that their contribution to the study will be confidential and the data provided can only be use for the purpose of the study. The respondents were also inform that no form of financial incentive will be given to them for being part of the study and that their choice of not being part of the study cannot stop them from getting any benefit that may come as a result of the study.

## Competing interests

 Authors declare that they have no competing interests.

## Authors’ contributions

 AIJ and PAB initiated the study. AIJ and KAB designed the study and provided training and guidance for data collection and analysis. AIJ and DL further prepared the original draft of the manuscript. PAB assisted with supervision, editing and technical support in all area of the study. AB and HMC assisted with the data collection and provide monitoring to entire team for data collection. Analysis was done by AIJ and IKD. PT contributed to the critical review of the first draft of the paper and also organized it in a format required by the journal. All authors made significant contributions to the final work. PAB and AIJ are guarantors.

## Funding

 This work was co-funded by the SLAM committee Canada and the authors.
